# Veterinary Comparative Pathology, a Scientific Tool for a Thriving Planet

**DOI:** 10.3390/ani13091504

**Published:** 2023-04-28

**Authors:** Alejandro Suárez-Bonnet, Gustavo A. Ramírez Rivero

**Affiliations:** 1 The Royal Veterinary College, University of London, London WC1E 7HU, UK; 2 Agrifood, Forestry and Veterinary Campus, Universitat de Lleida, 25003 Lleida, Spain

In recent years, Earth has overcome unpredictable challenges. From drastic climate change to a viral pandemic of probable zoonotic origin, these concurrent events have raised, to an unprecedented scale, the awareness that the natural world dominates humankind and not vice versa.

Where does veterinary pathology fit in this storm of events? More than a century ago, Virchow Robins, the son of a butcher and often seen as the founder of modern medicine and pathology, and other contemporary scientists like Robert Koch and Louis Pasteur, worked with an enviable mind on diseases that affected both animals and humans. William Osler, a human physician, also studied parasitic diseases in animals and named the parasite *Oslerus osleri* a nematode of canids. In fact, we owe the term ‘One Medicine’, the precursor of ‘One Health’, to Dr. Osler. As science used to be more open and scientists considered the natural world, human beings, and nonhuman animals as one, the concept of ‘One Medicine’ may have naturally developed. In contrast, current medical, biomedical, and basic research works focus on their individual areas, overlooking the bigger picture of the ‘One Health’ concept.

In this Special Issue, now edited as a book entitled *Comparative Pathology and Immunohistochemistry of Veterinary Species*, the reader can find pathology-focused, rigorously peer-reviewed manuscripts on different aspects of veterinary pathology from a comparative pathology perspective with a particular focus on immunohistochemistry as an ancillary diagnostic tool and a complementary technique in pathology research. 

As veterinary pathologists, we have broad knowledge of disease processes in various species with variable physiology and response to disease. The variety of areas where comparative veterinary pathology will elucidate the pathogenesis of vertebrate and invertebrate animal diseases is as wide as the number of diseases, aetiologies, and species known by science, whether they are neoplastic, infectious, non-infectious, or environmentally related. 

Comparative pathology, or as we prefer to call it, ‘One Pathology’, is the cornerstone of biomedical sciences and, ultimately, veterinary and human medicine. Only if we understand that there are no physical or philosophical boundaries between species, diseases, and the natural world and that they are, in reality, related and causally linked will we be able to unveil the secrets to a healthier planet.

